# Identification of an Immune-Related Gene Signature Based on Immunogenomic Landscape Analysis to Predict the Prognosis of Adult Acute Myeloid Leukemia Patients

**DOI:** 10.3389/fonc.2020.574939

**Published:** 2020-11-20

**Authors:** Ruiqi Zhu, Huishan Tao, Wenyi Lin, Liang Tang, Yu Hu

**Affiliations:** ^1^ Institute of Hematology, Union Hospital, Tongji Medical College, Huazhong University of Science and Technology, Wuhan, China; ^2^ Department of Gynecology and Obstetrics, Union Hospital, Tongji Medical College, Huazhong University of Science and Technology, Wuhan, China

**Keywords:** acute myeloid leukemia, immune-related gene signature, immunogenomic landscape, prognosis prediction, data mining

## Abstract

Acute myeloid leukemia (AML) is a hematopoietic malignancy characterized by highly heterogeneous molecular lesions and cytogenetic abnormalities. Immune disorders in AML and impaired immune cell function have been found to be associated with abnormal karyotypes in AML patients. Immunotherapy has become an alternative therapeutic method that can improve the outcomes of AML patients. For solid tumors, the expression patterns of genes associated with the immune microenvironment provide valuable prognostic information. However, the prognostic roles of immune genes in AML have not been studied as yet. In this study, we identified 136 immune-related genes associated with overall survival in AML patients through a univariate Cox regression analysis using data from TCGA-AML and GTEx datasets. Next, we selected 24 hub genes from among the 136 genes based on the PPI network analysis. The 24 immune-related hub genes further underwent multivariate Cox regression analysis and LASSO regression analysis. Finally, a 6 immune-related gene signature was constructed to predict the prognosis of AML patients. The function of the hub IRGs and the relationships between hub IRGs and transcriptional factors were investigated. We found that higher levels of expression of CSK, MMP7, PSMA7, PDCD1, IKBKG, and ISG15 were associated with an unfavorable prognosis of AML patients. Meanwhile, patients in the TCGA-AML datasets were divided into a high risk score group and a low risk score group, based on the median risk score value. Patients in the high risk group tended to show poorer prognosis [P = 0.00019, HR = 1.89 (1.26–2.83)]. The area under the curve (AUC) was 0.6643. Multivariate Cox Regression assay confirmed that the 6 IRG signature was an independent prognostic factor for AML. The prognostic role of the immune related-gene signature was further validated using an independent AML dataset, GSE37642. In addition, patients in the high risk score group in the TCGA dataset were found to be of an advanced age, IDH mutation, and M5 FAB category. These results suggested that the proposed immune related-gene signature may serve as a potential prognostic tool for AML patients.

## Introduction

Chemotherapy with or without stem cell transplantation has been used as the standard form of therapy for AML patients for many years. Despite advanced progression in anti-leukemic drug mining, the overall survival (OS) of AML patients remains unsatisfactory. The majority of patients die of relapse originating from minimal residual disease (MRD) (1). Thus, exploration of alternative therapeutic methods to eliminate MRD is imperative. Relapse occurs not only due to AML stem cell resistance to chemotherapy treatment, but also because AML cells are able to escape from immunosurveillance (2). Abnormal interactions between AML cells and the immune environment may result in AML cells evading immune control. For example, the immune molecule, KIR 2DL2, has been reported to be more frequently expressed in AML samples, indicating that it helps AML cells escape immunosurveillance (3). Additionally, AML cells in relapsed patients are always mutated and show resistance to NK cell-mediated lysis. On the surface of these mutated AML cells, co-stimulatory molecules have been frequently found to be downregulated (4). Immunotherapy has recently gained popularity as a therapeutic option for relapsed AML patients, through which specific immune cells are activated to kill cancer cells and immunosurveillance activity is re-established. Therefore, a conclusion can be drawn from previous studies that immune dysregulation plays an important role in AML relapse and that immune related genes may provide prognostic information for AML patients.

The most commonly used method to predict the prognosis of AML patients at present is the ELN genetic risk stratification system. However, the accuracy of the existing prognostic system has not been established as patients may show different outcomes and varying durations of survival. Along with the rapid development of genetic sequencing technology, prognostic signatures based on multiple gene integration, ncRNA integration, autophagy-related gene integration or immune-related gene (IRG) have been constructed and validated in various types if tumors (5–10). Some of these risk score signatures have shown great sensitivity and specificity in predicting the prognosis of cancer patients and may be used as potential tools for guiding individualized treatment.

IRG signatures for survival prediction have been validated in several types of cancers but a signature had not been developed for AML as yet. In this study, we identified 27 hub immune genes by analyzing prognostic immune landscape in AML using TCGA and GTEx databases. Next, we constructed a six IRG signature using 27 hub genes to predict the prognosis of AML patients. The signature was able to distinguish between patients with different OS and patients with a high risk score were always found to be associated with unfavorable clinical variants. The signature was further validated using the GSE37642 dataset. Our results provided new insights into the immune prognostic landscape, which can be used in further studies and has the potential to be used as a tool to predict survival of AML patients, in addition to the existing ELN risk stratification system.

## Materials and Methods

### Data Collection From Publicly Available Databases

RNA-seq data of 173 AML samples were downloaded from TCGA database (https://portal.gdc.cancer.gov/repository, TCGA-AML program). After the exclusion of 11 samples that did not include all relevant clinical data and 18 promyelocytic AML samples, a total of 143 AML samples were included in this study. Gene expression data and clinical data of the GSE37642 dataset were downloaded from GEO database (https://www.ncbi.nlm.nih.gov/geo/). The IRG list was obtained from the InnateDB database (http://www.innatedb.com/). Gene expression data and phenotype data of 192 normal whole blood samples were downloaded from the GTEx (https://www.gtexportal.org/home/) database. A total of 18,217 intersection genes were obtained using TCGA-AML and GTEx datasets, and were further analyzed. Clinical characteristics of patients in TCGA-LAML dataset were shown in **Table 1**.

**Table 1:** Clinical characteristics of patients in TCGA-LAML dataset.

Discrete variables	Number	Percentage (%)
Gender		
Male	78	54.55
Female	65	45.45
FAB subtype		
M0	15	10.49
M1	36	25.17
M2	35	24.48
M4	34	23.78
M5	18	12.59
M6	2	1.4
M7	3	2.1
Karyotype		
Favorable	17	11.89
Intermediate	94	65.73
Poor	30	20.98
Unknown	2	1.4
Living status		
Dead	97	67.83
Alive	46	32.17
IDH mutation		
Positive	27	18.88
Negative	114	79.72
Unknown	2	1.4
FLT3 mutation		
Positive	35	24.48
Negative	101	70.63
Unknown	7	4.9
RAS mutation		
Positive	8	5.59
Negative	132	92.31
Unknown	3	2.1
NPM1 mutation		
Positive	42	29.37
Negative	98	68.53
Unknown	3	
Continuous Variables	Range	Median
Age (years)	18–88	60
Overall survival (days)	0–2861	334
WBC (10^9/L)	1–297	18

### Identification of Differentially Expressed Genes Between AML and Normal Whole Blood Controls

Data processing was carried out using the R Bioconductor (Version 3.6.2) package. Normalization of data of the TCGA and GTEx datasets was conducted using the “normalize between array” function of the limma package. The differentially expressed genes (DEGs) between the AML samples and normal controls were identified using the DESeq2 package. Genes with a Log2 |Fold Change| of >2 and FDR (False Discovery Rate) of ≤0.05 were regarded as DEGs. Venn diagrams were constructed using the VennDiagram package to obtain AML-related IRGs.

### Bio-Functional Analysis of the AML-Related IRGs

The bio-functional analysis, which included Gene Set Enrichment Analysis (GSEA), Gene Ontology (GO) analysis and Kyoto Encyclopedia of Genes and Genomes (KEGG) pathway enrichment analysis, was conducted using the clusterProfiler and enrichplot packages. The cutoff P values and Q values for both the KEGG and GO analyses were 0.05 and the cutoff P value for the GSEA was 0.05.

### Identification of Hub Genes and Its Molecular Characteristics

A survival analysis was conducted on the AML-related IRGs. A Univariate Cox regression analysis was conducted and the PPI (Protein-Protein Interaction) network of the survival associated IRGs was constructed based on PPI data obtained from the STRING databases (a confidence score of >700 was used for network construction). Nodes with the top 5% of degrees were identified as hub IRGs. The PPI network was displayed using Cytoscape software (Version 3.7.2). Forest plots of the differentially expressed hub IRGs were depicted using the forestplot package in R.

To identify the molecular characteristics of the hub IRGs, the relationships between TFs and hub genes were determined based on data from TRANSFAC (http://gene-regulation.com/pub/databases.html). Heatmaps were created using the pheatmap package in R.

### Survival Analysis and IRG Signature Construction

A multivariate Cox regression analysis was carried out on the hub IRGs and clinical variates, including age, WBC, and karyotype, were used to select IRGs that were independently associated with OS in AML. LASSO regression analysis was used after the multivariate Cox analysis to further identify candidate genes that could be used in the risk score signature. Survival analysis was conducted using R Bioconductor. The survival package was used for the univariate and multivariate Cox regression analyses, while the glmnet package was used for the LASSO regression analysis.

### Statistical Analysis

The Kaplan-Meier curve was used to show differences in OS between the high-risk group and low-risk group patients, based on the IRG signature. The AUC of the ROC curve was calculated using the survival ROC package. Chi-square test was used to determine correlation between the risk score and clinical variants. All statistical analyses were conducted using R software. A P value of < 0.05 was considered to indicate significance.

## Results

### Differentially Expressed IRGs in AML

**Figure 1:**
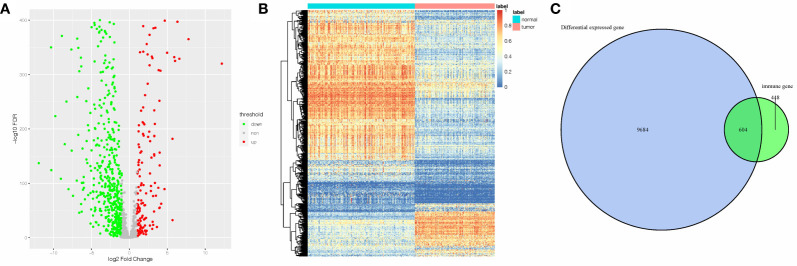
A total of 605 AML-related IRGs are shown in the **(A)** volcano plot, **(B)** heatmap, and **(C)** AML-related IRGs.

The DEGs between 143 AML samples obtained from TCGA database and 192 normal whole blood controls from GTEx database were identified. A total of 10,288 DEGs were identified. Among them, 4,781 genes were upregulated, and 5,507 genes were downregulated. Next, we extracted the IRGs from InnateDB database and the intersections between the extracted IRGs and DEGs were determined. A total of 604 AML-related IRGs were identified (**Figure 1C**), while 138 of these were upregulated and 466 were downregulated. The AML-related IRGs are shown in **Figures 1A, B**. Detailed information of AML-related IRGs is given in **Supplementary Table 1**.

### Bio-Functional Analysis of the AML-Related IRGs

**Figure 2:**
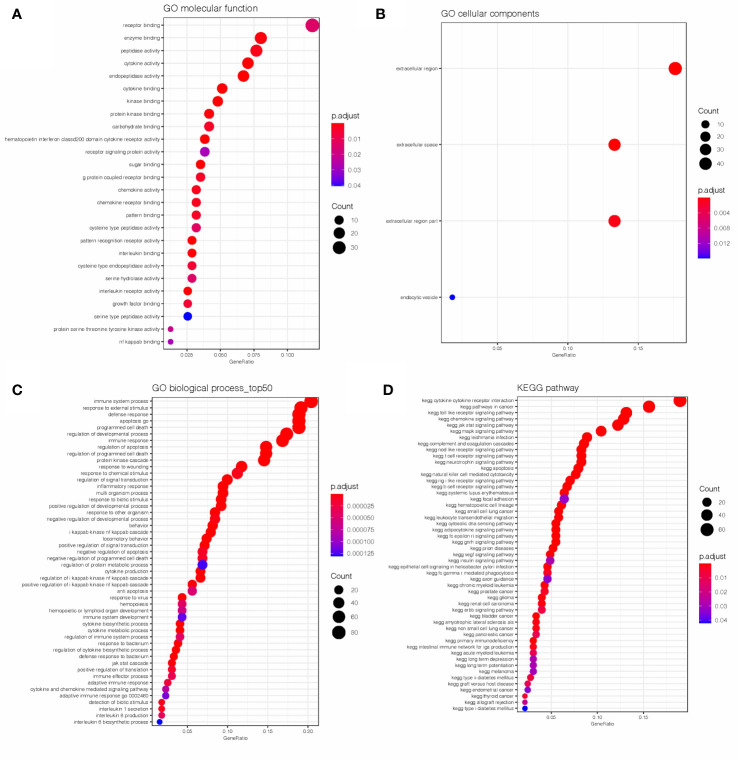
Results of the GO **(A–C)** and KEGG **(D)** analyses of AML-related IRGs.

GO and KEGG analyses were conducted on the 604 IRGs to investigate the biological function of the AML-related IRGs. As shown in **Figures 2A–C**, for the GO analysis, in terms of molecular functions (MFs), these genes were most enriched in receptor binding, cytokine activity, receptor signaling protein activity and interleukin binding. In terms of biological processes (BPs), these genes were most enriched in immune system process, apoptosis and programmed cell death. In terms of cellular components (CCs), these genes were most enriched in the extracellular region. As expected, the KEGG analysis (**Figure 2D**) found that the AML-related IRGs were enriched in immune pathways, such as the cytokine-cytokine receptor interaction pathway, Toll-like receptor pathway, Jak-Stat signaling pathway and chemokine signaling pathway.

### Identification of AML-Related IRGs Associated With OS

**Figure 3:**
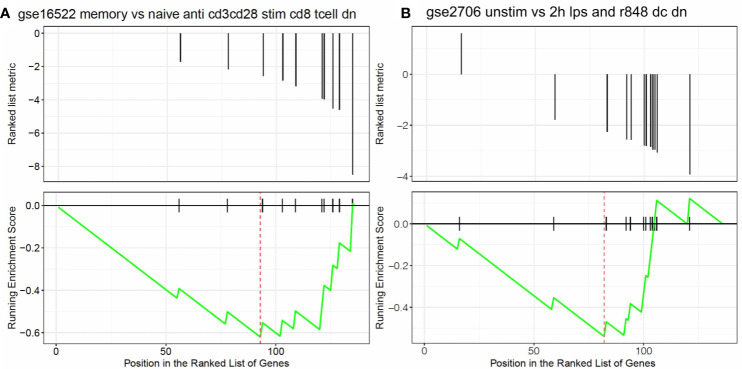
Results of the GSEA. Survival related IRGs were enriched in immunological pathways, especially in pathways associated with the differentiation of **(A)** naïve B cells vs neutrophils and **(B)** naïve CD8 T cells vs monocytes.

The univariate Cox regression analysis found that 136 AML-related IRGs were associated with OS (**Supplementary Table 2**). GSEA was carried out to explore the pathways that the survival related IRGs were enriched in. Significant enrichment was found in 5 immunological pathways (**Supplementary Table 3**), especially in pathways involved in the immunologic gene sets (**Figures 3A, B**).

### Identification of Hub IRGs and Their Molecular Characteristics

**Figure 4:**
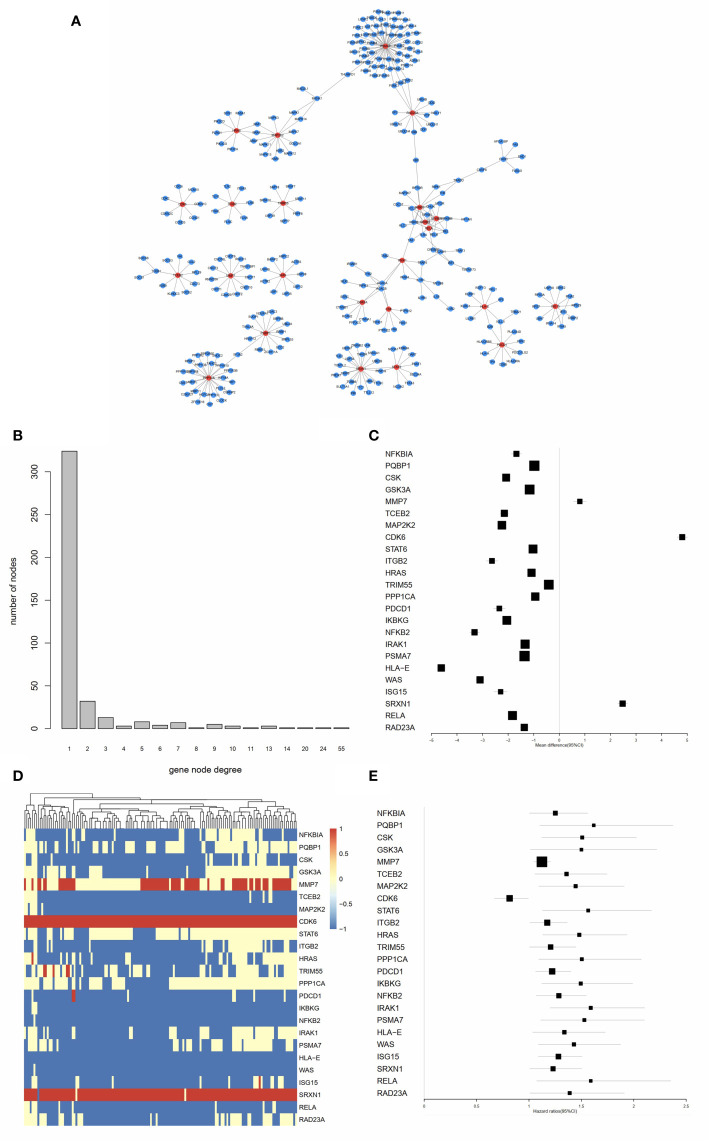
Identification of hub IRGs and their molecular characteristics. **(A)** PPI network of the 24 hub IRGs. **(B)** The relationship between gene node degree and the number of nodes. **(C)** Mean expression differences of hub IRGs between AML and normal controls. **(D)** Cluster graph of 24 hub IRGs in the AML samples. The darker red color represents a higher level of gene expression, while the darker blue color represents a lower level of gene expression. **(E)** Hazard ratios (HR) show the prognostic value of the hub IRGs. A Gene with a HR of greater than 1 indicates a risk factor for prognosis, while a HR of less than 1 indicates a protective effect.

As shown in **Figure 4A**, the PPI network of the IRGs was constructed to identify hub IRGs. Among the 136 survival related IRGs, those with the top 5% of degrees in the network were regarded as hub IRGs. In total, 24 hub IRGs were identified. The relationship between gene node degree and the number of nodes is shown in **Figure 4B**. The mean expression difference of the hub IRGs between the AML and normal controls is shown in **Figures 4C, D**. CDK6, MMP7, and SRXN1 were upregulated in AML samples, while all other genes were downregulated. The hazard ratios indicate the prognostic value of hub IRGs and are shown in **Figure 4E**. The results indicated that dysregulation of most hub genes was related with an unfavorable prognosis. The mutation analysis found that only six genes were mutated in the five samples. Detailed information on these mutations are provided in **Supplementary Table 4**.

### Molecular Mechanisms of the Hub IRGs

**Figure 5:**
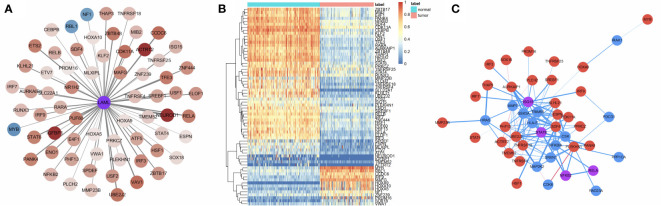
Molecular mechanisms of the hub IRGs. **(A)** 66 TFs were found to be associated with OS in AML patients. **(B)** Gene expression of the 66 TFs in the AML samples and normal controls. **(C)** The regulatory network between the 24 hub IRGs and the 66 TFs.

Transcriptional Factors (TFs) are of great importance in determining degrees in the PPI network for hub IRG identification. Thus, we analyzed the interactions between survival-related TFs and hub IRGs to identify the molecular mechanisms of the hub IRGs. The univariate Cox regression analysis found that 66 TFs were associated with OS in AML (**Figure 5A**). Gene expression of the 66 TFs in AML samples and normal controls are shown using a heatmap (**Figure 5B**). The regulatory network was constructed between the 24 hub IRGs and 66 TFs (**Figure 5C**). Pearson’s correlation coefficient was calculated and was used as the weight of each gene in the network. A correlation coefficient of more than 0.4 was set as the cutoff value.

### Prognostic Signature Construction Using Hub IRGs

**Figure 6:**
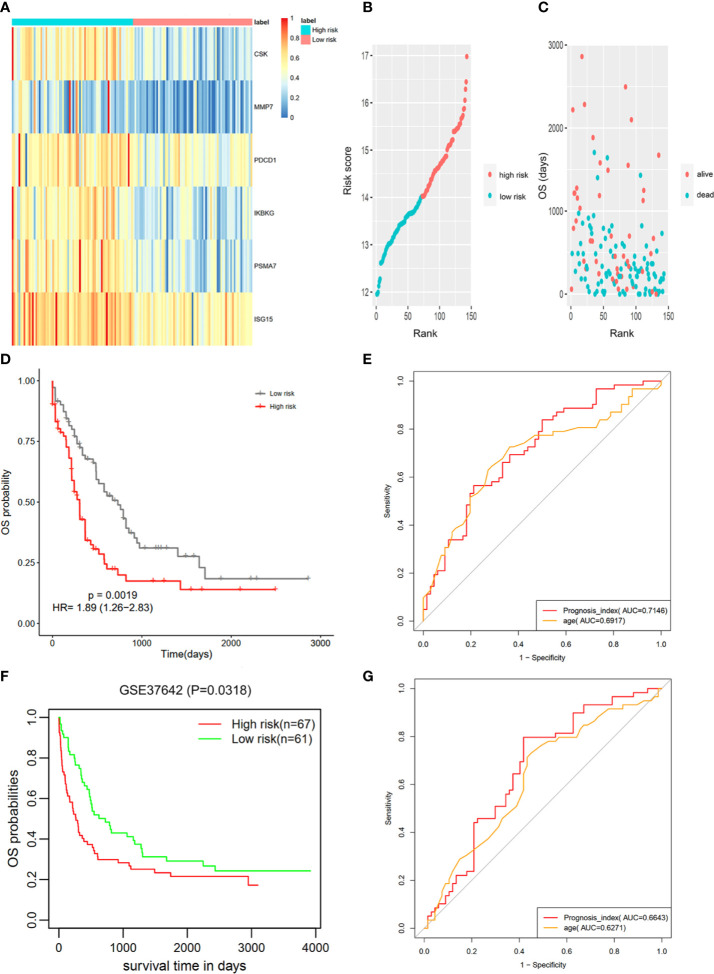
Construction of the 6 IRG signature. **(A)** The expression of 6 IRGs in the high and low risk score group is shown using a heatmap. **(B, C)** The relationship between the risk score, OS and living status. **(B)** Patients were ranked based on risk score. **(C)** Higher risk score was found to be associated with more deaths and shorter OS. **(D)** Based on data from TCGA database, the Kaplan-Meier curve showed that patients in the high risk group exhibited significantly shorter OS than patients in the low risk group [P = 0.00019, HR = 1.89 (1.26–2.83)]. **(E)** The AUC of the signature was 0.7146. **(F)** The signature was further validated using an independent GEO dataset (GSE37642). The Kaplan-Meier curve demonstrated that patients in the high risk score group exhibited significant shorter OS than those in the low risk score group. **(G)** The AUC was 0.6643.

**Figure 7:**
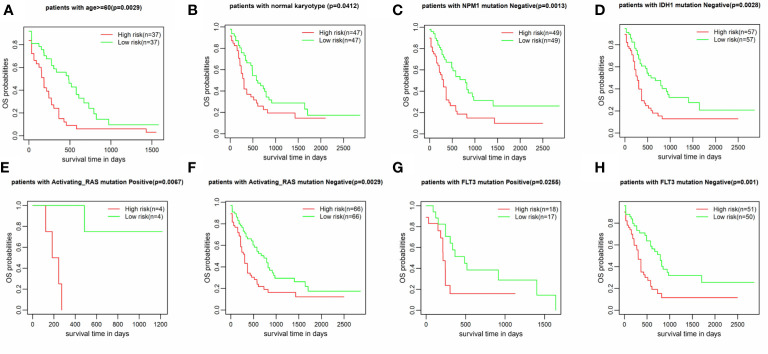
Stratified survival analysis according to clinical factors and genetic lesions. **(A)** age >60, **(B)** Normal karyotype **(C)** NPM1 mutation negative, **(D)** IDH1 mutation negative, **(E, F)** RAS mutation positive/negative, **(G, H)** FLT3 mutation positive/negative.

The IRG prognostic signature was constructed using hub IRGs using the results of the multivariate and LASSO regression analyses and 6 IRGs were identified. The expressions of the 6 IRGs in the high and low risk score groups is shown using a heatmap (**Figure 6A**). The formula used for the calculation of the risk score signature was as follows: Risk score = 0.3827 * expression of CSK+ 0.1383 * expression of MMP7 + 0.3114 * expression of IKBKG + 0.1589 * expression of PDCD1 + 0.3812 * expression of PSMA7 + 0.2127 * expression of ISG15. To evaluate the performance of the signature, we divided patients into a high risk group and low risk group, based on the median risk score. The relationship between the risk score and living status is shown in **Figures 6B, C**. As shown in **Figure 6D**, Kaplan-Meier curve was used to demonstrate that patients in high risk group had significantly shorter OS than patients in low risk group [P = 0.00019, HR = 1.89 (1.26–2.83)]. The ROC curve showed that the AUC of the risk score signature was 0.7146, indicating that the IRG signature had a moderate potential for survival prediction in AML (**Figure 6E**). To validate the prognostic value of the 6 IRG signature in an independent dataset, we utilized the GSE37642 dataset. The clinical information of GSE37642 was shown in **Table 2**. As shown in **Figure 6F**, the Kaplan-Meier curve demonstrated that patients in the high risk score group had a significantly shorter OS than those in the low risk score group. The AUC was 0.6643 (**Figure 6G**), indicating good performance of the IRG signature. Age and molecular genetic characteristics may have an impact on the prognosis. Therefore, we conducted a further stratified survival analysis for age (grouped with a median of 60 years), four molecular genetic characteristics (IDH mutation, FLT3 mutation, NPM1 mutation, activating RAS mutation) and karyotype risk. We found that the prognostic model can further distinguish the prognosis of patients with/without RAS mutation, patients with/without FLT3 mutation, patients over 60 years of age, patients with normal karyotype, patients without IDH mutation and patients without NPM1 mutation (**Figures 7A–H**), which makes up for the gap that the existing clinical prognostic models for such patients cannot be further distinguished. For patients under 60, patients with IDH mutation positive and patients with NPM1 mutation positive, no significant differences were observed in OS (data not shown). To validate the prognostic signature is an independent prognostic factor for AML, we conducted multivariate Cox regression assay using the risk score and clinical factors which were important for AML prognosis. The results showed that after adjustment for the known prognostic parameters, higher risk score of the 6 IRG signature was still a predictor for poor OS in AML patients (**Table 3**).

**Table 2:** Clinical variables in patients of GSE37642.

Discrete Variables	Number	Percentage (%)
FAB subtype		
M0	8	6.25
M1	29	22.66
M2	47	36.72
M4	17	13.28
M5	19	14.84
M6	7	5.47
M7	1	0.78
Living Status		
Dead	95	74.22
Alive	33	25.78
Continuous Variables	Range	Median
Age (years)	18–85	60
Overall Survival (days)	2–3919	402

**Table 3:** Multivariate Cox Regression assay for OS using known clinical prognosticators and 6 IRG risk score.

			95% Exp(B) CI	
Variable	P-value	Exp(B)	Lower	Upper
Age	0.001	3.428	2.061	5.701
Gender	0.646	0.892	0.548	1.452
WBC	0.587	0.872	0.532	1.429
FAB category	0.395	1.071	0.914	1.256
Karyotype type	0.031	1.734	1.142	2.963
IDH1 mutation	0.287	0.721	0.395	1.317
FLT3 mutation	0.041	1.759	1.022	3.026
RAS mutation	0.879	1.08	0.404	2.883
NPM1 mutation	0.454	0.82	0.488	1.378
6 IRG score	0.003	2.097	1.277	3.444

Exp(B): The exponentiation of the B coefficient. Older age, poor karyotype, FLT3 positive and high 6 IRG score were associated with Exp(B)>1.

### Relationship Between the 6 IRG Signature and Clinical Factors

**Figure 8:**
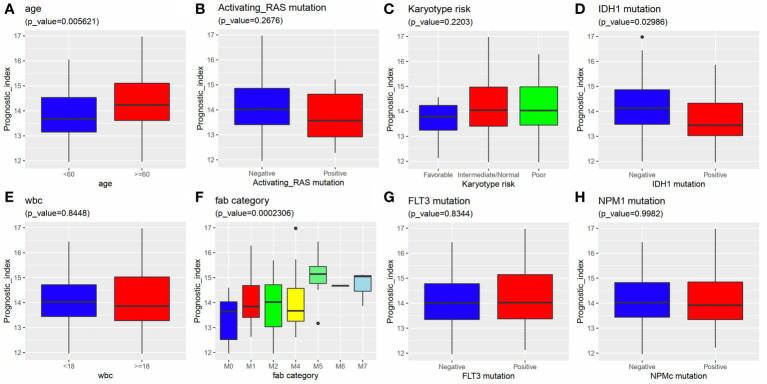
Relationship between the 6 IRG signature and clinical factors. A higher risk score was associated with **(A)** an advanced age, **(D)** IDH1 mutation and **(F)** FAB category. The risk score had nothing to do with **(B)** activating RAS mutation, **(C)** Karyotype, **(E)** WBC, **(G)** FLT3 mutation and **(H)** NPM1 mutation.

We further investigated the relationship between the 6 IRG signature and clinical variants, including age, gender, WBC, karyotype, and FAB category using data from the TCGA-AML dataset. The results showed that a higher risk score was always associated with an advanced age, IDH mutation, and M5 FAB category. No differences were observed between the risk score and gender, karyotype, RAS mutation, FLT3 mutation, NPM1 mutation, and WBC (**Figures 8A–H**).

## Discussion

During recent years, the cancer immune landscape has been found to be of vital importance in precision medicine for both solid tumors and hematological malignancies (11–14). This can be attributed to the deep understanding of tumor microenvironment obtained during the past few decades. Emerging evidence has proved the efficacy of the clinical use of immune checkpoint inhibitors (ICBs), such as the anti–PD-1 drug, nivolumab, and the anti–CTLA-4 drug, ipilimumab. These drugs have been used to treat metastatic melanoma (15), renal cell carcinoma (16), and non-small cell lung cancer (17). However, not all patients can benefit from immunotherapeutic agents. Their effects depend on the phenotype of the tumor microenvironment and the expression of various immune genes. Patients with T cell-inflamed tumors are more likely to respond to ICBs, while tumors without adaptive immune cell infiltration are likely to be resistant to immunotherapy (18). Therefore, elucidation and an understanding of the immune landscape of tumors may not only provide insights into tumor immune dysregulation, but may also lay the foundation for overcoming immunotherapy resistance.

Great effort has been made to investigate tumor immune cell infiltration and to identify immune genes of prognostic value for solid tumors (19–21). Patients with AML are able to achieve durable remission following “adoptive immunotherapy”, which is also known as allogeneic stem cell transplantation (22). The immune microenvironment is of vital importance for AML initiation and progression (23). Recent years, some immune prognosticators have been identified (24–26). For example, SIG3 genes GZMB and FoxP3 are reported to be individual predictors for shorter OS in AML patients (26). IFN-γdominant tumor microenvironment (TME) predicts shorter OS for AML patients. In addition, although IFN-related gene sets improve the prediction for chemotherapy resistance, another study suggests that IFN-γdominant TME is associated with good response to flotetuzumab (27). However, studies have not been conducted as yet to collectively evaluate immune status and develop novel IRG signatures that can improve prognosis prediction of AML patients. In this study, we identified differentially expressed IRGs and conducted a bioinformatics analysis. We identified hub IRGs in AML by constructing the PPI network. Mutation status, prognostic values, as well as interactions between hub IRGs and TFs were investigated to describe the immune landscape of AML. A six IRG signature was constructed using the results of multivariate and LASSO cox regression analyses. The patients in high risk score group tend to have poor prognosis, indicating that the signature successfully predicted the prognosis of AML patients. In addition, high risk score was associated with unfavorable clinical factors, including an advanced age, high WBC and poor karyotypes.

KEGG analysis showed that the differentially expressed IRGs were involved in cytokine-cytokine receptor interaction and signaling pathways, such as JAK/STAT pathway, MAPK pathway and Toll-like receptor pathway, which are closely associated with cancers. Additionally, GO BP results showed that these IRGs were significantly involved in cancer related processes, including immune system process, apoptosis, programmed cell death and signaling transduction. Our results were consistent with previous findings, which showed that dysregulation of immune genes and the immunosuppressive microenvironment were indispensable for leukemia development (28–30).

Survival related IRGs and hub IRGs were identified as described in the Results section. Next, the relationship between TFs and hub IRGs were investigated. Many of the most significant TFs were found to be involved in the progression of the disease and were able to predict the prognosis of AML patients. For example, ETS2 was found to be a downstream target of both the PI3K/AKT and Ras/Raf/MAPK pathways. Dysregulation of these pathways were closely associated with AML progression. Liu et al. reported that the high level of expression of ETS2 predicted the unfavorable prognosis for AML patients (31). ENO1 functions as a glycolytic enzyme and was found to be upregulated in the AML samples (32). HSF1 is a transcriptional regulator of heat shock response, which has been found to promote AML progression by activating Wnt-β-catenin pathway (33). These findings support our results that the hub IRGs identified were closely associated with immune dysregulation and tumor progression in AML.

Among the six IRGs included in the signature, IKBKG and PSMA7 have not been studied in AML as yet, while CSK, MMP7, PDCD1, and ISG15 have been reported in AML. CSK (c-Src, C-Terminal Src Kinase) is a regulator of Src family kinases and has been found to be crucial for T-cell activation. The complex formed between TrkA and c-Src has been detected in leukemia, while the inhibition of c-Src suppressed the Akt/mTOR pathway (34). MMP7 (Matrix metalloproteinases 7) is closely associated with angiogenesis, which is involved in regulating the body’s sensitivity to chemotherapy (35). MMP-7 was found to be upregulated in patients with AML, especially in patients who had relapsed (36). Wu et al. also suggested that higher levels of expression of MMP7 can predict poor OS in AML (37). PDCD1 (PD-1, Programmed Cell Death 1 Protein) has been found to be highly expressed in many cancers and was found to be responsible for the escaping of cancer cells from immunosurveillance. Upregulation of surface PD-1 leads to T cell inactivation. Studies have shown that after chemotherapy in AML patients, the number of suppressor T cells and the activity of PD-1/PD-L1 increase significantly. These results suggest that PCDC1 reduces chemotherapy sensitivity, and the use of checkpoint inhibitors can enhance the effect of chemotherapeutic drugs (38–40). In AML, higher PD-1 expression was found in all T cell subpopulations (CD4 T effector and regulatory cells and CD8 T cells) in untreated and relapsed patients (41). Besides, PD-L1 is related to adaptive immune resistance and is predictive with the efficacy of pembrolizmab in clinical application (42, 43). PD-1 scores identified in previous study also improved the prediction of therapeutic resistance after cytarabine and anthracycline treatment in both childhood and adult AML patients (26). Additionally, it has been shown that the group with the highest levels of PD-L1 expression in AML had the worst prognosis (41). These findings are consistent with our conclusions that high levels of PD-1 expression is indicator of poor prognosis in AML. ISG15 was found to have played an important role in ATRA-induced differentiation of NB4 cells. Knockdown of ISG15 attenuated the differentiation promoting effect of ATRA and inhibited ISGylation (44). Besides, ISG15 is a member of IFN-related DNA damage resistance signature in breast cancer and it is related to chemotherapy resistance, suggesting that high expression of ISG15 is a result of chronic activation of IFN signaling (45). This result was contrary to the conclusion found through this study that high levels of ISG15 expression was related to poor prognosis, suggesting that the ISG15 gene may perform additional functions, which need to be confirmed in subsequent studies.

In conclusion, a 6 IRG signature was constructed to predict the prognosis of AML patients based on immune landscape analysis. The signature successfully stratified patients into a high risk group and a low risk group based on mean OS. At the same time, poor clinical prognostic factors, including an advanced age, high WBC and poor karyotypes, were often found among high risk patients. The AML immune landscape analysis identified novel AML targets and signaling pathways, while the signature provided a novel method of predicting prognosis.

## Data Availability Statement

The datasets presented in this study can be found in online repositories. The names of the repository/repositories and accession number(s) can be found in the article/**Supplementary Material**.

## Author Contributions

RZ and HT carried out the data analysis. RZ and WL prepared the manuscript. RZ, LT, and YH had substantial contributions to the conception and design of the work. HT, YH, and LT revised the manuscript critically. YT and LT did final approval of the version to be published. All authors contributed to the article and approved the submitted version.

## Funding

YH: National Natural Science Fund, No. 31620103909. LT: Chen-Guang Project of Wuhan Science and Technology Bureau, 2017050304010276.

## Conflict of Interest

The authors declare that the research was conducted in the absence of any commercial or financial relationships that could be construed as a potential conflict of interest.

## Supplementary Material

Supplementary Figure 1
**(A)** Relationship between the 6 IRG signature and the expression of TP53 in AML patients. **(B)** Relationship between the 6 IRG signature and DNMT3A mutation status in AML patients.
