# Supplementary figures and images for: Identification of an Immune-Related Gene Signature Based on Immunogenomic Landscape Analysis to Predict the Prognosis of Adult Acute Myeloid Leukemia Patients
Source: Front Oncol. 2020 Nov 20;10:574939. doi: 10.3389/fonc.2020.574939 (PMC7714942; doi:10.3389/fonc.2020.574939)

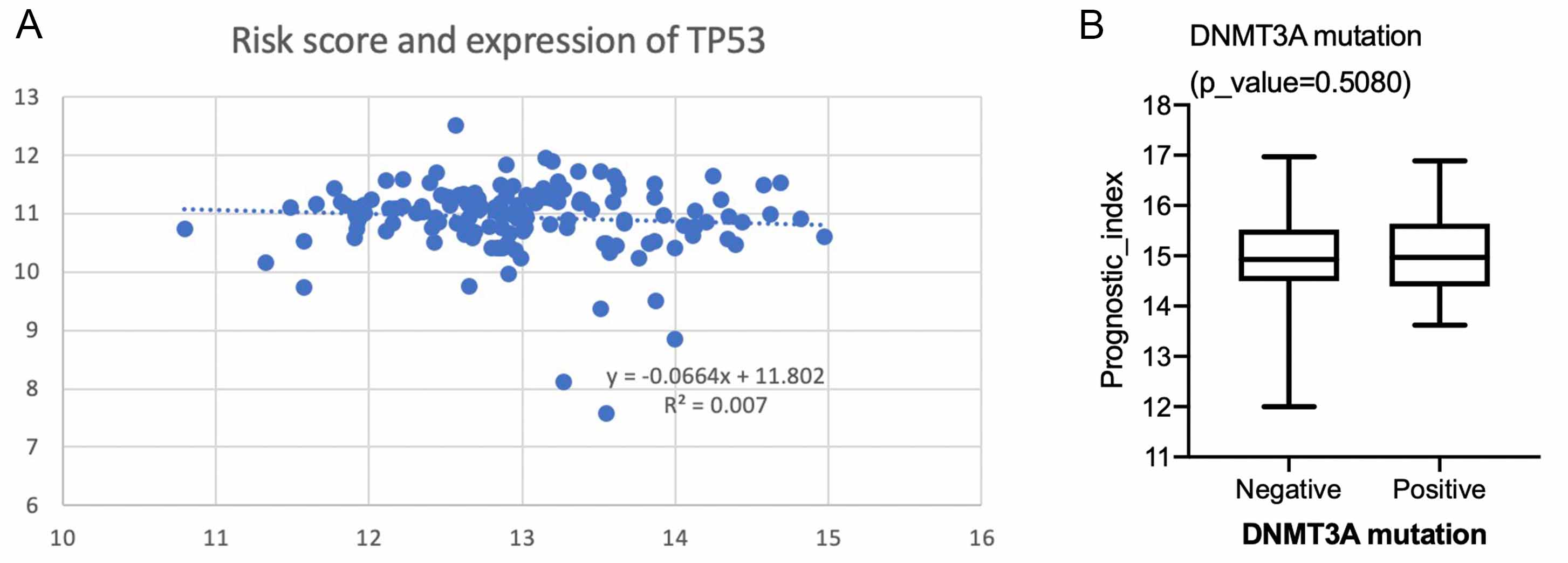

Supplement: Supplementary Figure 1 — (A) Relationship between the 6 IRG signature and the expression of TP53 in AML patients. (B) Relationship between the 6 IRG signature and DNMT3A mutation status in AML patients. [file Image_1.jpeg]
